# Simulated Epilepsy

**Published:** 1868-07-15

**Authors:** 


### CHICAGO MEDICAL JOURNAL.

*Vol. XXV.--JULY* 15, 1868.—*No.* 14.

### SIMULATED EPILEPSY.

*From Les Annales de l'Hygiene el de Médecine Légale.*

TRANSLATED EXPRESSLY FOR THE CHICAGO MEDICAL JOURNAL BY WALTER HAY, M.D., POST OFFICE BLOCK, CHICAGO.

CERTAIN simulators of epileptic attacks acquire so great facility of imitation, that expert alienists themselves can be, and have been, frequently deceived. Such examples are not rare.

Inspired by the labors of M. Brown-Séquard upon the disturbance of the great sympathetic in epileptic attacks, M. Auguste Voisin has thought that this disturbance would be extended throughout the vaso-motor system, and consequently to the arteries of the limbs, and has instituted upon several patients, upon healthy persons who had taken violent exercise, and upon simulators of epilepsy, sphygmographic experiments, which have conducted him to the following conclusions:

1. Epileptic seizures and simple (epileptic) vertigo induce disturbances of the arterial circulation, which may be recognized by means of the sphygmograph of Marcy, and which are characterized by very decided curves, then by ascending lines of great height, and a very marked dicrotism, which persists from half an hour to several hours.

2. These sphygmographic forms have been obtained from my patients, and from myself after gesticulation, violent efforts, or rapid running.

3. The study of the pulse of an epileptic simulator has demonstrated to me the absolute absence of resemblance between the sphygmographic diagrams formed under these conditions, and those taken from epileptics.

4. Being given an epileptic simulator, it will suffice to submit him to regular observation, and to take several tracings (sphygmographic) during an hour after the attacks, in order to decide the question of simulation.

The experiments of M. Auguste Voisin are very interesting, and the means of diagnosis which they appear to indicate may be very valuable. It is desirable that they should be repeated and confirmed by other observers, and that the new field of observation which they open should not remain uncultivated.

